# Scrotal Myiasis in a Child Due to Cordylobia anthropophaga

**DOI:** 10.7759/cureus.59417

**Published:** 2024-04-30

**Authors:** Majed H Wakid, Yasser S Sharafeldein, Angham A Almakki, Dhuha A Alidrisi, Abeer A Bashinim

**Affiliations:** 1 Medical Laboratory Sciences, Faculty of Applied Medical Sciences, King Abdulaziz University, Jeddah, SAU; 2 Special Infectious Agents Unit, King Fahd Medical Research Center, Jeddah, SAU; 3 Department of Laboratory and Blood Bank, Security Forces Hospital, Makkah, SAU; 4 Department of Pediatric Infectious Diseases, Security Forces Hospital, Makkah, SAU

**Keywords:** case report, maggots, cordylobia anthropophaga, myiasis, scrotal, child

## Abstract

Human myiasis is caused by the invasion of tissue or organs by maggots of certain dipterous flies. The present case is of an eight-year-old boy complaining of painful swelling in the scrotum with localized discharge. A maggot was removed and sent to a specialized laboratory for identification. The case was diagnosed as a scrotal myiasis caused by *Cordylobia anthropophaga*. The wound was cleaned with antiseptics, then antibiotic treatment was applied. Two days later, the wound healed completely. All previously documented cases of scrotal myiasis were associated with *Dermatobia hominis*. We document here the first case of scrotal myiasis in children caused by *C. anthropophaga,* and the necessity to raise awareness of myiasis among health professionals.

## Introduction

Myiasis is the invasion of the tissues or organs of a vertebrate host by maggots of flies belonging mainly to the families *Calliphoridae *and *Sarcophagidae*. However, other families of flies can also cause myiasis [1]. Myiasis could be external or internal, and the invasion of the maggot could be obligatory, facultative, and sometimes accidental. Obligatory myiasis is a serious parasitism in which the larvae infest and destroy living tissues or organs, while in facultative myiasis the larvae mainly infest decomposing tissues. Accidental myiasis occurs when certain flies lay eggs or deposit maggots accidentally on exposed organs and tissues or, in rare cases, by ingestion. In addition, myiasis can be classified according to the parts of the body affected, for example, cutaneous, ophthalmic, nasopharyngeal, intestinal, or urogenital [2]. The severity of symptoms depends on the species of flies, the number of maggots, and organs or tissues affected.

## Case presentation

An eight-year-old boy, who was not known to have any previous medical illness, presented in February 2023 with a three-day history of scrotal swelling that was progressive and associated with pain and discomfort. The patient began experiencing purulent discharge from a localized area in the scrotum. He had no history of fever or shock but had pain during urination. On examination, the child appeared healthy but was in pain. Local examination of the genitals showed generalized swelling throughout the scrotum, accompanied by redness and tenderness, and there was also a small punctum discharging a little pus. The mother revealed that the family lived in the countryside, and there was contact with trees, and many types of animals and insects.

**Figure 1:**
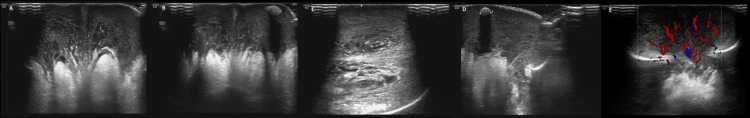
Ultrasound of the scrotum. (A-D) Diffuse edema of the scrotal wall and swelling; (E) Increased vascularity on Doppler interrogation.

Ultrasound of the scrotum showed diffuse edema of the scrotal wall and swelling, up to 13 mm in diameter with an increased vascularity on Doppler interrogation. A tiny calcific focus was noted in the left scrotal wall. Multiple inguinal lymph nodes were noted, likely reactionary in nature and associated with regional echogenic fat smudging. It was clear that both testicles had not descended into the deep portion of their right inguinal canal, and ultimately no collection was observed (Figure 1).

Blood testing was reassuring, including a normal complete blood count (CBC) and inflammatory markers (C-reactive protein and procalcitonin). Urinalysis and urine culture were negative. The swab taken from the pus for culture also showed no bacterial growth.

The patient was admitted to the pediatric ward as a case of scrotal cellulitis. He started parenteral antimicrobial treatment with amoxicillin-clavulanic acid for one day, then upgraded to ceftriaxone and clindamycin as his condition progressed. The pediatric surgery team was consulted, and the case was followed up. Four days after admission, the patient showed improvement in swelling and signs of inflammation, but there was a small area of localized swelling that did not show improvement, with little drainage, although the ultrasound showed no visible collection and calcification. The pediatric surgery team participated in the case, and the decision was to transfer the child to the operating room for examination. A small incision was made in the most prominent area, and a live larva was observed, and then extracted (Video 1). Accordingly, the case was diagnosed as scrotal myiasis with secondary scrotal cellulitis.

**Video 1:** The extracted maggot during the operation in the surgery room.

For further conclusive species identification, the extracted maggot was sent to the diagnostic parasitology laboratory at the Special Infections Agents Unit, King Fahd Medical Research Center in Jeddah, Saudi Arabia, and cultured in soil as previously reported [3]. The size and microscopic morphology of the larva, and adult fly were consistent with *Cordylobia anthropophaga* [1,4].

The patient's condition improved two days after the maggot removal. No signs of inflammation were found during the examination, and the patient was discharged home.

Signed informed consent was obtained from the patient’s mother (legal guardian) for publication of this case report.

## Discussion

It is known that *C. anthropophaga* is an obligatory myiasis fly belonging to the family *Calliphoridae*. This tumbu fly invades man and a wide range of reservoir hosts, especially rodents and domestic dogs. Females are attracted to urine or may oviposit on underclothes [1,2,4].

To the best of our knowledge, we report the first case of scrotal myiasis in children due to *C. anthropophaga*. This fly is commonly associated with furuncular myiasis, but is rare in Saudi Arabia [5-16]. All cases of scrotal or penile myiasis previously reported in Turkey, the United States, Brazil, and India were mainly in adult patients due to infestation by *Dermatobia hominis* botfly [17-20].

**Figure 2:**
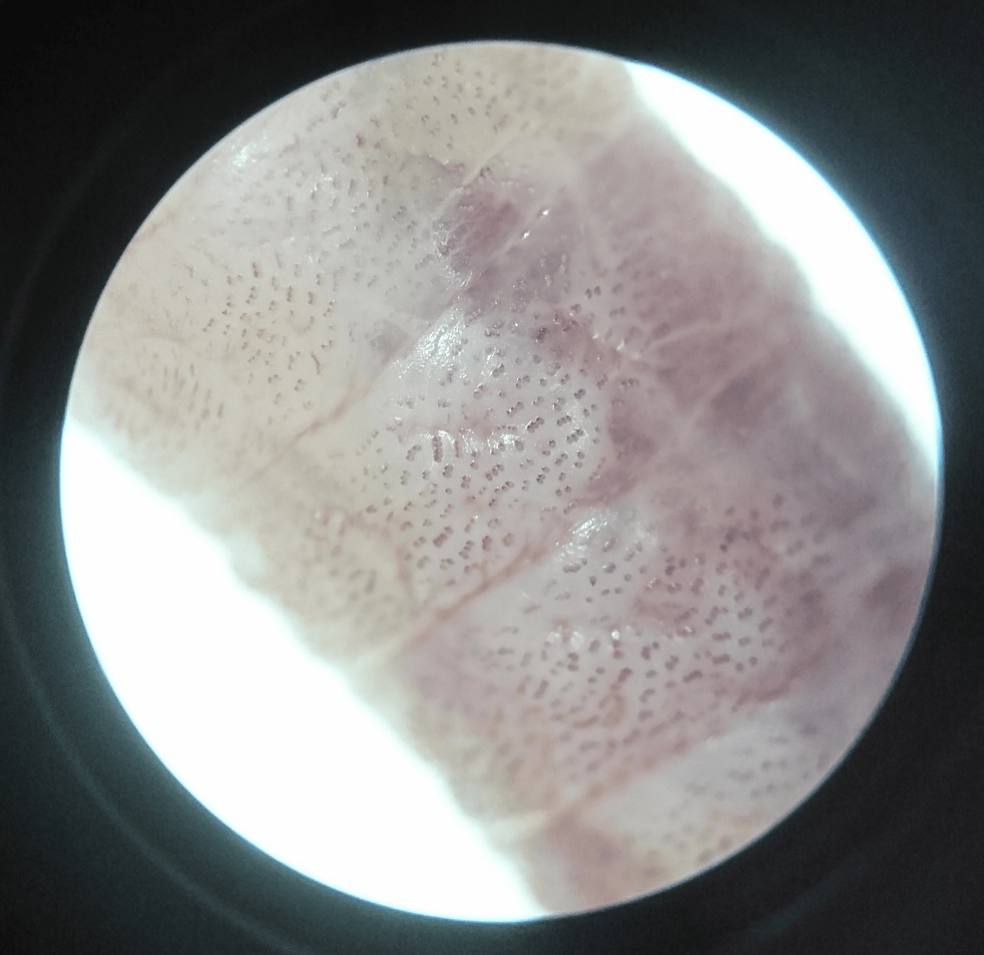
Microscopic appearance shows the spines on the body of the extracted maggot.

**Figure 3:**
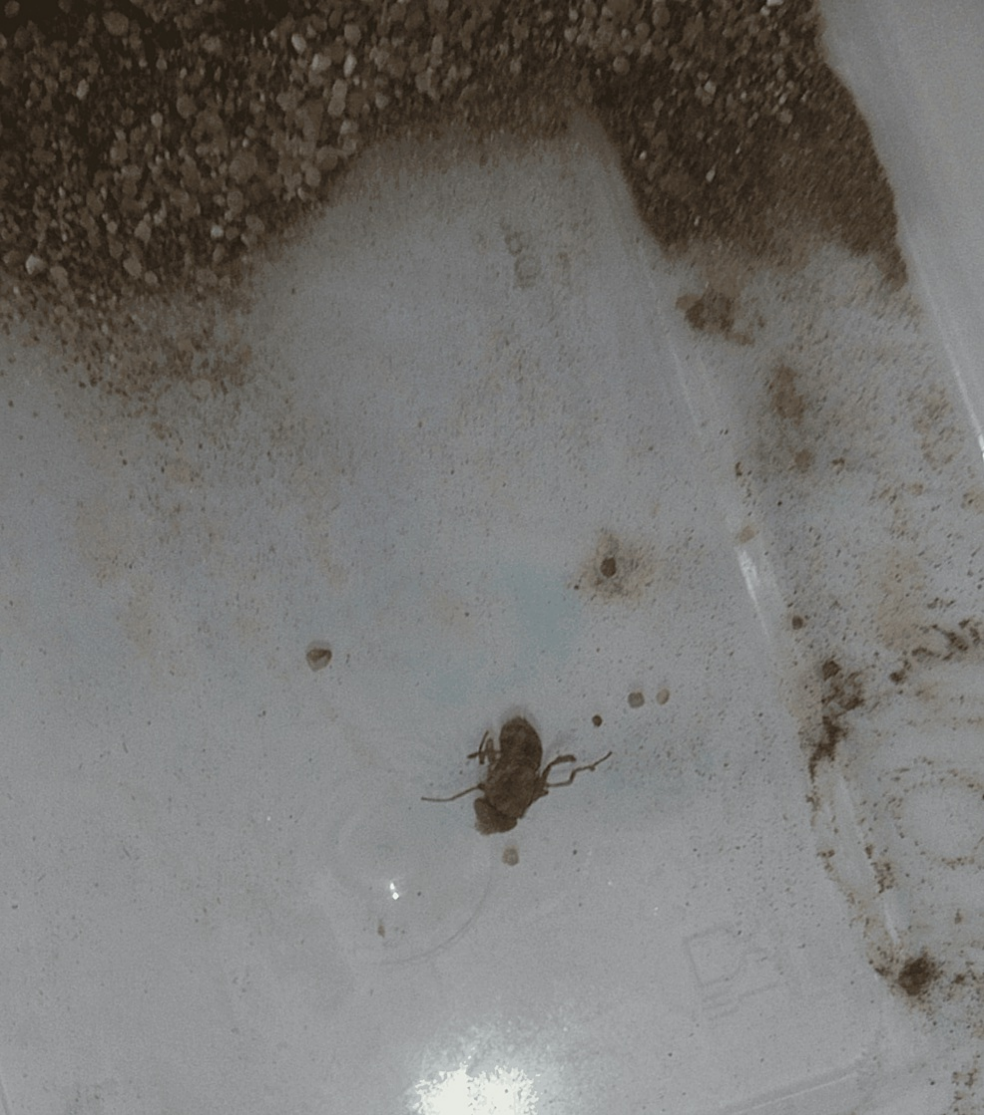
The emerged adult fly after culturing in soil for 12 days.

We identified *C. anthropophaga* based on the morphological features of the isolated larva (yellowish-white, about 12 mm, covered with numerous spines that are grouped into three or more transverse rows per segment, and the posterior spiracles have a weakly sclerotized peritreme and three sinuous slits), and the adult fly that emerged after culture for 12 days (about 10 mm long, light-brown in color, with two broad dorsal longitudinal thoracic stripes) (Video 2) (Figure 2, 3).

**Video 2:** The extracted maggot in the culturing container before addition of soil.

The child was hospitalized for surgery to remove the larva and treatment with antibiotics. The pain and discomfort associated with the present case may be due to the larva burrowing deep into the tissues and feeding with its mouth hooks.

To prevent human myiasis, general improvement of sanitation, personal hygiene, and fly control with insecticides are needed. It is important to avoid contact with flies and animals surrounded by insects. Clothes should be washed well with hot water, dried away from insects, and ironed thoroughly [1,4].

Effective treatment of any type of myiasis, including the present scrotal case, is achieved primarily by the physical removal of all larvae and the use of topical antiseptics and oral antibiotics to prevent secondary infections or complications [3].

## Conclusions

This case revealed that human scrotal myiasis, including in children, can be caused by *C. anthropophaga*, and cases of human myiasis are not rare but still neglected. We recommend community educational programs about myiasis and how to prevent and control it. Awareness should also be raised among health professionals on how to recognize, identify, and treat myiasis cases.
